# Roles of Testosterone and Estradiol in Mediation of Acute Neuroendocrine and Electroencephalographic Effects of Sevoflurane During the Sensitive Period in Rats

**DOI:** 10.3389/fendo.2020.545973

**Published:** 2020-09-30

**Authors:** Ningtao Li, Ning Xu, Yunan Lin, Lei Lei, Ling-Sha Ju, Timothy E. Morey, Nikolaus Gravenstein, Jiaqiang Zhang, Anatoly E. Martynyuk

**Affiliations:** ^1^ Department of Anesthesiology and Perioperative Medicine, Henan Provincial People’s Hospital, People’s Hospital of Zhengzhou University, Zhengzhou, China; ^2^ Department of Anesthesiology, University of Florida College of Medicine, Gainesville, FL, United States; ^3^ McKnight Brain Institute, University of Florida College of Medicine, Gainesville, FL, United States

**Keywords:** estradiol, testosterone, neonatal anesthesia, sevoflurane, corticosterone

## Abstract

Testosterone (T), predominantly acting through its derivative 17β-estradiol (E2), regulates the brain’s sexual differentiation in rodents during the perinatal sensitive period, which mirrors the window of vulnerability to the adverse effects of general anesthetics. The mechanisms of anesthesia’s adverse effects are poorly understood. We investigated whether sevoflurane alters T and E2 levels and whether they contribute to sevoflurane’s acute adverse effects in postnatal day 5 Sprague-Dawley rats. The rats underwent electroencephalography recordings for 2 h of baseline activity or for 1 h before and another hour during 2.1% sevoflurane exposure, followed by collection of trunk blood and brain tissue. Pharmacological agents, including the GABA type A receptor inhibitor bicuculline and the aromatase inhibitor formestane, were administered 30 min before sevoflurane anesthesia. Sevoflurane increased serum T levels in males only. All other effects of sevoflurane were similar in both sexes, including increases in serum levels of E2, hypothalamic mRNA levels of aromatase, estrogen receptor α (*Er*α) [not estrogen receptor β (*Erβ)*], Na^+^-K^+^-Cl^−^ cotransporter (*Nkcc1*)/K^+^-Cl^−^ cotransporter (*Kcc2*) mRNA ratio, electroencephalography-detectable seizures, and stress-like corticosterone secretion. Bicuculline and formestane alleviated these effects, except the T level increases. The ERα antagonist MPP, but not the ERβ antagonist PHTPP, reduced electroencephalography-detectable seizures and normalized the *Nkcc1/Kcc2* mRNA ratio. Collectively, sevoflurane exacerbates levels of T in males and E2 in both sexes during the period of their organizational effects in rodents. Sevoflurane acts through GABA_A_R-mediated, systemic T-independent elevation of E2 to cause electroencephalography-detectable seizures, stress-like corticosterone secretion, and changes in the expression of genes critical for brain development.

## Introduction

According to 2016 World Health Organization estimates, the number of surgeries performed globally rose from 226.4 million in 2004 to 312.9 million in 2012 (1). This progress would not be possible without modern general anesthesia, which can be viewed as a state of pharmacologically induced “reversible brain coma” (2). Despite advancements in refining anesthesia approaches, multiple studies support the idea that the effects of general anesthetics, which may also act as endocrine disruptors and stressors, are not completely reversible upon anesthesia withdrawal. The adverse effects of general anesthesia at the beginning and the end of the lifespan are an increasingly recognized health concern and the subject of extensive clinical and laboratory research (3–7). Experimental evidence indicates that GABAergic anesthetic agents, such as propofol, isoflurane, or sevoflurane, administered to neonatal rats, acutely induce electroencephalography (EEG)-detectable seizures and increased systemic levels of the main stress hormone corticosterone (8–10). The long-term effects of these anesthetics comprise an abnormal (increased) hypothalamic and hippocampal Na^+^-K^+^-Cl^−^ cotransporter (*Nkcc1*)/K^+^-Cl^−^ cotransporter (*Kcc2*) mRNA ratio, an exacerbated hypothalamic-pituitary-adrenal (HPA) axis responses to stress, and behavioral deficiencies (8, 9, 11–13).

Intracellular concentrations of Cl^−^, the main charge carriers through GABA type A receptor (GABA_A_R) channels, are elevated in many neurons in the neonatal brain because of the relatively high and low levels of the NKCC1 and KCC2 Cl^−^ co-transporters, respectively (14, 15). Activation of GABA_A_R in these neurons causes Cl^−^ efflux, a strong membrane depolarization or excitation, and Ca^++^ influxes through the voltage-gated Ca^++^ channels and Ca^++^-permeable N-methyl-D-aspartate receptor channels (14). GABA_A_R-initiated depolarization and related Ca^++^ influxes regulate a wide spectrum of biological processes (14). The magnitude of GABA_A_R excitatory signaling and the proper timing of its transition from excitatory to inhibitory are key for normal brain development and functioning (14). Delays/impairments in the transition to inhibitory GABA_A_R signaling have been linked in humans and animal models to several cognitive neuropsychiatric disorders, such as autism spectrum disorder, schizophrenia, and Rett syndrome (16–18). Inhibition of the NKCC1 activity at the time of neonatal anesthesia ameliorates anesthetic-caused EEG-detectable seizures and many of the long-term developmental effects of GABAergic anesthetics in rats, suggesting that anesthetic-exacerbated excitatory GABA_A_R signaling at the time of anesthesia can be an initial step in the pathways that mediate the developmental effects of GABAergic anesthetics (8–12).

We have recently presented indirect evidence that the primary female sex steroid hormone 17β-estradiol (E2) may play a crucial role in mediating sevoflurane-caused EEG-detectable seizures by enhancing excitatory GABA_A_R signaling in neonatal rats (10). E2, however, is known to modulate GABAergic signaling not only by affecting the GABA_A_R-based neurotransmission machinery (10, 19–22), but also by altering the expression and activity of KCC2 and NKCC1, respectively (23, 24). In the neonatal rodent brain, E2 exerts the brain’s sexual differentiation (masculinization) through organizational/persistent actions during the sensitive period, which mirrors the window of vulnerability to the adverse effects of general anesthetics. In rats, the sensitive period starts on embryonic days 18.5 to 19.5, with the onset of testosterone (T) production in the testis and ends during the second postnatal week when exogenous sex hormones can no longer induce brain masculinization in females (25). E2 in the brain is produced by converting testis-derived T in males and *via de novo* synthesis in the brain in both sexes. In both pathways, the final step in E2 synthesis is T aromatization by the enzyme aromatase, whose activity is regulated by Ca^++^ (26). E2 acts primarily through its receptors α (ERα) and β (ERβ) (27–29). It is widely accepted that neonatal rodents become less susceptible to the neurodevelopmental effects of general anesthetics during the second postnatal week (13, 30–32).

Here, we studied the roles of T and E2 in the mediation of sevoflurane’s acute (initial) adverse effects in the postnatal day (P) 5 rats. We did so by measuring sevoflurane-caused EEG-detectable seizures, changes in systemic levels of T, E2, and corticosterone, and changes in the expression of hypothalamic *aromatase*, *Erα*, *Erβ*, *Nkcc1*, and *Kcc2* under different treatment conditions to modulate GABA_A_R and T/E2 signaling pathways. T/E2 may affect sexual differentiation in the rodent brain during this age period through lasting organizational effects (25) and sevoflurane may induce long-term developmental abnormalities (8, 9, 11, 12). For that reason, understanding the involvement of sevoflurane-altered levels of T and E2 in mediating the acute adverse effects of sevoflurane in neonatal rats at the time of anesthesia may help to explain the mechanistic basis of more complex long-term deficiencies induced by the anesthetic.

## Materials and Methods

### Animals

The University of Florida Institutional Animal Care and Use Committee approved all experimental procedures. Sprague-Dawley rats were bred at the University of Florida animal care facility. We housed the rats under controlled illumination (12-h light/dark cycle, lights on at 7:00 a.m.) and temperature (23–24°C) with free access to food and water. Within 24 h of delivery, litters were culled to 12 pups. Pups from each litter were used for different treatment conditions. Multiple sets of animals were used in the experiments.

### Electroencephalography, Anesthesia Regimen, and Treatment Groups

**Figure 1:**
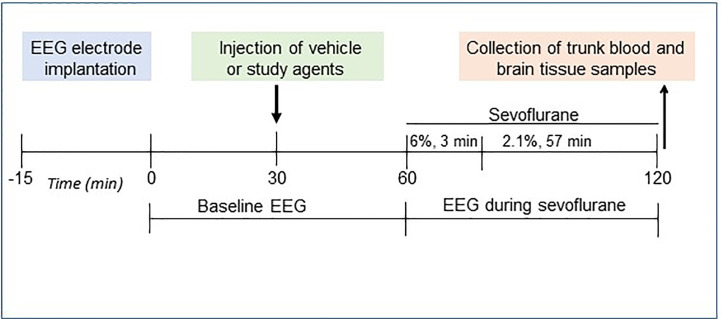
Study design.

The P5 rats were instrumented for EEG recording during a minor 12- to 15-min surgical procedure performed under isoflurane anesthesia (2.0%–2.5%) as we previously described (10, 30, 33). The four EEG electrodes were implanted bilaterally in the occipital and frontal regions of the rat’s skull, with the left frontal electrode serving as the reference electrode. The EEG recordings were started after the rats recovered from isoflurane anesthesia for electrode implantation; they lasted for 2 h of baseline activity (group 1, the Control group) or for 1 h before the initiation of sevoflurane anesthesia (baseline activity) and continued for another hour during sevoflurane anesthesia: 6% sevoflurane for 3 min for anesthesia induction and 2.1% sevoflurane for 57 min for anesthesia maintenance (groups 2–7; **Figure 1**). We have previously verified that no obvious differences in the effects of sevoflurane to cause EEG-detectable seizures were detected when EEG electrode implantation was done either prior or 1 to 2 days before the start of EEG recording (34). During the EEG recordings, the rats were in a thermostated chamber to maintain body temperature at ~37°C with a continuous supply of 30% oxygen in air (1.5 L min^−1^). A rectal temperature probe was placed in some animals to monitor body temperature. Gas monitoring was performed using a calibrated Datex side stream analyzer (Datex-Ohmeda, Helsinki, Finland), which sampled from the animal chamber interior. According to Orliaguet et al. (35), 2.1% sevoflurane lies near the 0.6 minimum alveolar concentration for P5 rats. At 2.1% sevoflurane, the pups did not exhibit a righting reflex but responded to a noxious stimulation. None of the animals exhibited cyanosis and all were breathing regularly during anesthesia exposure. Previously, we have shown that blood glucose and gas levels after 2.1% sevoflurane anesthesia were in the normal range (30). The EEG recordings were performed using an EEG/electromyogram system (Pinnacle Technology, Lawrence, KS, United States). Acquisition of the EEG was performed using Sirenia software (Pinnacle Technology). The sampling interval per signal was 200 μs (5 kHz). Sirenia Score (Pinnacle Technology), Clampfit 9.2 (Axon Instruments, Union City, CA, United States), and Mini Analysis (Synaptosoft Inc., Fort Lee, NJ, United States) programs were used for EEG data analysis. Data were filtered offline using a bandpass Bessel (8-pole) 0.04- to 56-Hz filter. EEG patterns characterized by an amplitude of at least three times higher than baseline and rhythmic activity (>2 Hz) that lasted for at least 3 s and abruptly reverted to the baseline were defined as seizure-like EEG patterns. In most cases, these patterns started as high frequency-low amplitude activity that developed to increased amplitude and decreased frequency and then abruptly reverted to baseline activity. All parameters for EEG seizures such as the total duration, number of episodes, and average episode duration were calculated for the entire 60-min period of sevoflurane exposure. All EEG records were analyzed by three independent investigators. The investigators who analyzed the EEG records were blinded to the experimental conditions. Animals that exhibited episode(s) of seizure-like EEG patterns before the start of anesthesia were removed from the data analysis. Typically, in our studies about 5% of animals exhibit episode(s) of seizure-like EEG patterns before the start of anesthesia and are not included in the data analysis (10, 30, 33). Histopathological analysis links most of these seizures to brain injuries during surgery for the EEG electrode implantation.

Rats were randomized for treatment groups using a randomization plan generator (http://www.randomization.com/) and the investigators were blind to group assignments. Treatments or vehicle were administered to the P5 rat pups 30 min prior to sevoflurane anesthesia. The following treatment groups were investigated:

1. (Group 1) EEG recording for 2 h without exposure to sevoflurane (the Control group);
2. (Group 2) EEG recording for 1 h without sevoflurane and for 1 h with sevoflurane; vehicle [subcutaneous (SC) or intraperitoneal injection (IP)] 30 min prior to the start of anesthesia with sevoflurane (the Vehicle + Sevo group);
3. (Group 3) E2 synthesis inhibitor formestane (2 mg/kg, SC) 30 min prior to sevoflurane anesthesia (the Formestane + Sevo group);
4. (Group 4) GABA_A_R antagonist bicuculline methiodide (0.01 mg/kg, IP) 30 min prior to sevoflurane anesthesia (the Bicuculline + Sevo group);
5. (Group 5) ERα antagonist MPP (0.2 mg/kg, IP) 30 min prior to sevoflurane anesthesia (the MPP + Sevo group);
6. (Group 6) ERβ antagonist PHTPP (4 mg/kg, IP) 30 min prior to sevoflurane anesthesia (the PHTPP + Sevo group);
7. (Group 7) G-protein-coupled estrogen receptor (GPER) antagonist G-15 (5 μg/kg, IP) 30 min prior to sevoflurane anesthesia (the G-15 + Sevo group).

All study agents were dissolved in 33% DMSO plus 67% saline, which was the vehicle noted in Group 2. Therefore, equal volumes of DMSO and saline were used as vehicle. DMSO at these doses does not cause any detectable effects on hyperexcitatory EEG or serum corticosterone levels in neonatal rats (33). These concentrations/doses were chosen based on our published and preliminary findings and data in the literature (10, 30, 33, 36). Immediately after we completed EEG recordings, rats were sacrificed by decapitation while being anesthetized with sevoflurane and trunk blood and brain tissue samples were isolated.

### Tissue Collection

After decapitation, the trunk blood samples were collected and centrifuged at 4°C, 1,000 g for 15 min, and kept at −80°C for hormone assays. The brains were removed from the skull onto ice pads. The hypothalamus was isolated by making an anterior cut at the level of the optic chiasm, a posterior coronal section anterior to the mammillary bodies, two sagittal cuts parallel to the lateral ventricles, and a dorsal horizontal cut at the level of the anterior commissure, as described previously (37). All tissue samples were placed in vials filled with RNAlater solution (Invitrogen, Carlsbad, CA, United States) and stored at −80°C.

### Measurements of Serum T, E2, and Corticosterone Levels

Using commercially available kits and following the manufacturer’s instructions, we measured serum levels of hormones in trunk blood samples isolated from P5 rats immediately after we completed EEG recording. Serum T, E2, and corticosterone concentrations were measured using ELISA kits (582701, Cayman Chemical Company, Ann Arbor, MI, United States; EA100859, Origene Technologies Inc., Rockville, MD, United States; and 501320, Cayman Chemical Company, respectively).

### Quantitative mRNA Measurements

We analyzed levels of mRNA for *aromatase*, *Erα*, *Erβ*, *Nkcc1*, and *Kcc2* in the hypothalamus *via* reverse transcription-PCR (qRT-PCR) in a StepOnePlus™ Real-Time PCR System (Applied Biosystems, Foster City, CA, United States) as previously described by our laboratory (12). We extracted RNA from the samples using an RNeasy Plus Kit (Qiagen, Valencia, CA, United States), reverse transcribed with a high-capacity cDNA reverse transcription kit (Bio-Rad Laboratories, Hercules, CA, United States), and analyzed *via* qRT-PCR. Taqman probes specific for the above genes were obtained from Applied Biosystems (Carlsbad, CA): *aromatase* (Rn00567222_m1), *Erα* (Rn01430446_m1), *Erβ* (Rn00562610_m1), *Nkcc1* (Rn00582505_m1), and *Kcc2* (Rn00592624_m1). Data were normalized to glyceraldehyde-3-phosphate dehydrogenase (*Gapdh*) mRNA (Rn01775763_g1). Gene expression was calculated using the ΔΔCT method and data are presented as relative fold change from control animals.

### Drugs

Sevoflurane (NDC: 0074-4456-04) was manufactured by AbbVie Inc. (North Chicago, IL, United States). The aromatase inhibitor formestane (CAS Number: 566-48-3) and the GABA_A_R inhibitor bicuculline methiodide (CAS Number: 40709-69-1) were purchased from Sigma-Aldrich (St. Louis, MO, United States). GPER antagonist G-15 [(3aS,4R,9bR)-4-(6-bromo-1,3-benzodioxol-5-yl)-3a,4,5,9b-tetrahydro-3H-cyclopenta[c]quinolone] (CAS Number: 1161002-05-6) was purchased from Cayman Chemical Company (Ann Arbor, MI, United States). The ERα antagonist MPP (1,3-Bis(4-hydroxyphenyl)-4-methyl-5-[4-(2-piperidinylethoxy)phenol]-1H-pyrazole dihydrochloride) (CAS Number: 289726-02-9) and the ERβ antagonist PHTPP (4-[2-phenyl-5,7-bis(trifluoromethyl)pyrazolo[1,5-a]-pyrimidin-3-yl]phenol) (CAS Number: 805239-56-9) were acquired from Santa Cruz Biotechnology (Santa Cruz, CA, United States).

### Statistical Analysis

We conducted statistical analyses on raw data using SigmaPlot 14.0 software (Systat Software Inc., San Jose, CA, United States), which automatically checks if a data set meets test criteria (Shapiro-Wilk for normality test and Brown-Forsythe for equal variance test). Values are reported as mean ± SEM. Animal numbers in each experiment are presented in the respective figure legends. An independent t-test was used to compare sevoflurane-induced EEG seizures in male and female rat pups and the effects of G-15 on sevoflurane-induced EEG seizures and serum levels of corticosterone. Two-way ANOVA with treatment and sex as independent variables was used to analyze EEG seizures, levels of T, E2, and corticosterone, and mRNA levels for *aromatase*, *Er*α, *Er*β, *Nkcc1*, and *Kcc2* in the control and sevoflurane-exposed male and female rat pups. One-way ANOVA was used to analyze the effects of pretreatments with bicuculline, formestane, MPP, and PHTPP on sevoflurane-induced changes in EEG seizures, serum levels of T, E2, and corticosterone, and mRNA levels of *aromatase*, *Er*α, *Er*β, *Nkcc1*, and *Kcc2* in each sex. All multiple pairwise comparisons were done with the Holm-Sidak method. *P* < 0.05 was considered statistically significant. Statistical details are presented in the text and in figure legends. The sample sizes in this study were based on previous experience with the same animal model, experimental techniques, and measured variables (8–12, 30, 33).

## Results

### Effects of Sevoflurane in Male and Female Rat Pups

**Figure 2:**
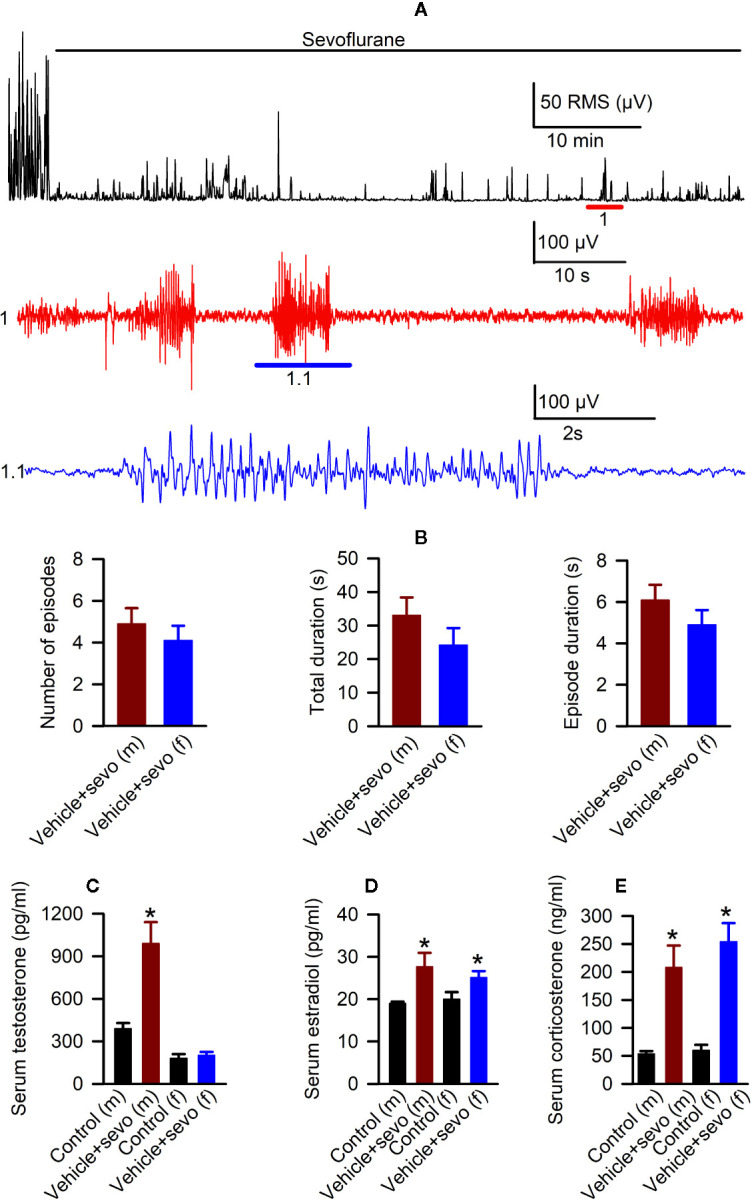
Effects of sevoflurane in postnatal day 5 rats at the systemic level. **(A)** Illustration of the electroencephalogram (EEG)-detectable seizures in a male rat pup during sevoflurane anesthesia. Top trace: root mean square (RMS) of the EEG of a male rat before and during exposure to sevoflurane. The red horizontal line marks the occurrence of EEG-detectable seizures (1); corresponding section of the EEG is shown at expanded time scale in 1 (red trace); 1.1 (blue trace) shows at expanded scale the section of EEG recording marked by blue horizontal line in 1. **(B)** Histograms showing parameters of EEG-detectable seizures during 60-min exposure to sevoflurane of male (*n* = 10) and female (*n* = 9) rats. Plots showing the levels of serum testosterone **(C)**, estradiol **(D)**, and corticosterone **(E)** in trunk blood samples collected from male and female rats immediately after completion of EEG recordings during 2 h of baseline activity (the Control group) or 1 h of baseline activity followed by another hour during exposure to sevoflurane (the Vehicle + Sevo group). Data are means ± SEM from six rats/group. *P < 0.05 vs. the Control group.

**Figure 3:**
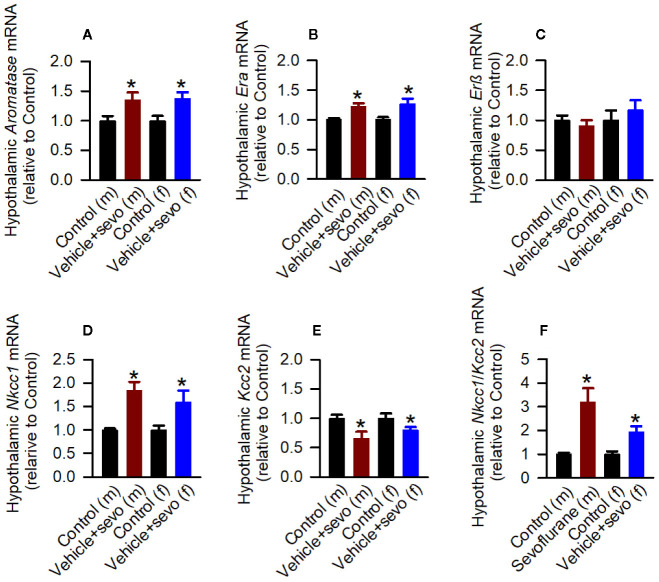
Effects of sevoflurane in postnatal day 5 rats at the molecular level. Brain tissue samples were collected from male and female rats immediately after completion of electroencephalography (EEG) recordings during 2 h of baseline activity (the Control group) or 1 h of baseline activity followed by another hour during exposure to sevoflurane (the Vehicle + Sevo group). **(A–F)** The respective levels of *aromatase* mRNA, estrogen receptor α (*Er*α) mRNA, estrogen receptor β (*Er*β) mRNA, Na^+^-K^+^-Cl^−^ cotransporter *(Nkcc1)* mRNA, K^+^-Cl^−^ cotransporter (*Kcc2*) mRNA, and *Nkcc1/Kcc2* mRNA ratio in the hypothalamus of male and female rats. Data normalized against control are means ± SEM from six rats/group. *P < 0.05 vs. the Control group.

Episodes of epileptic seizures were found in the electroencephalograms of male and female P5 rats recorded during 1 h of sevoflurane anesthesia (**Figures 2A, B**). The differences in the number of episodes of EEG-detectable seizures (t_(17)_ = 0.764; *P* = 0.455; **Figure 2B**), the total duration of seizures during 1 h of anesthesia (t_(17)_ = 1.205; *p* = 0.245; **Figure 2B**), and the duration of a single episode (t_(17)_ = 1.165; *P* = 0.260; **Figure 2B**) were not significant between the sexes. Immediately after we completed EEG recordings, the blood and brain tissue samples were collected to analyze serum levels of T, E2 and corticosterone and mRNA levels of *aromatase*, *Erα*, *Erβ*, *Nkcc1*, and *Kcc2* in the hypothalamus. Two-way ANOVA revealed significant effects of treatment and significant treatment x sex interaction on serum levels of T. Anesthesia with sevoflurane increased serum levels of T in male pups only (**Table 1**, **Figure 2C**). In contrast to serum levels of T, both male and female pups anesthetized with sevoflurane had similarly increased serum levels of E2 (**Table 1**, **Figure 2D**), serum levels of corticosterone (**Table 1**, **Figure 2E**), and hypothalamic mRNA levels of *aromatase*, *Er*α, *Nkcc1*, and the *Nkcc1*/*Kcc2* mRNA ratio, while hypothalamic mRNA levels of *Kcc2* were decreased in both sexes. Anesthesia with sevoflurane did not affect hypothalamic mRNA levels of *Er*β (**Table 1**, **Figures 3A–F**).

**Table 1:** Acute effects of sevoflurane in male and female P5 rat pups.

Measured variables	The results of the statistical analyses	
	Treatment	Treatment/sex interaction
Serum corticosterone	F_(1,20)_ = 44.290, *p* < 0.001	F_(1,20)_ = 0.575, *p* = 0.457
Serum testosterone	F_(1,20)_ = 15.366, *p* < 0.001	F_(1,20)_ = 13.348, *p* = 0.002
Serum estradiol	F_(1,17)_ = 10.318, *p* = 0.005	F_(1,17)_ = 0.689, *p* = 0.418
*Aromatase* mRNA	F_(1,20)_ = 13.083, *p* = 0.002	F_(1,20)_ = 0.006, *p* = 0.938
*Er*α mRNA	F_(1,19)_ = 18.886, *p* < 0.001	F_(1,19)_ = 0.098, *p* = 0.757
*Er*β mRNA	F_(1,19)_ = 0.0859, *p* = 0.773	F_(1,19)_ = 0.942, *p* = 0.344
*Nkcc1* mRNA	F_(1,20)_ = 19.093, *p* < 0.001	F_(1,20)_ = 0.563, *p* = 0.462
*Kcc2* mRNA	F_(1,20)_ = 11.595, *p* = 0.003	F_(1,20)_ = 0.740, *p* = 0.400
*Nkcc1*/*Kcc2* mRNA	F_(1,20)_ = 19.435, *p* < 0.001	F_(1,20)_ = 2.790, *p* = 0.110

Aromatase, aromatase gene; Erα, estrogen receptor α gene; Erβ, estrogen receptor β gene; Nkcc1, Na^+^-K^+^-Cl^−^ cotransporter gene; Kcc2, K^+^-Cl^−^ cotransporter gene.

### Effects of Pretreatments With Formestane and Bicuculline

**Figure 4:**
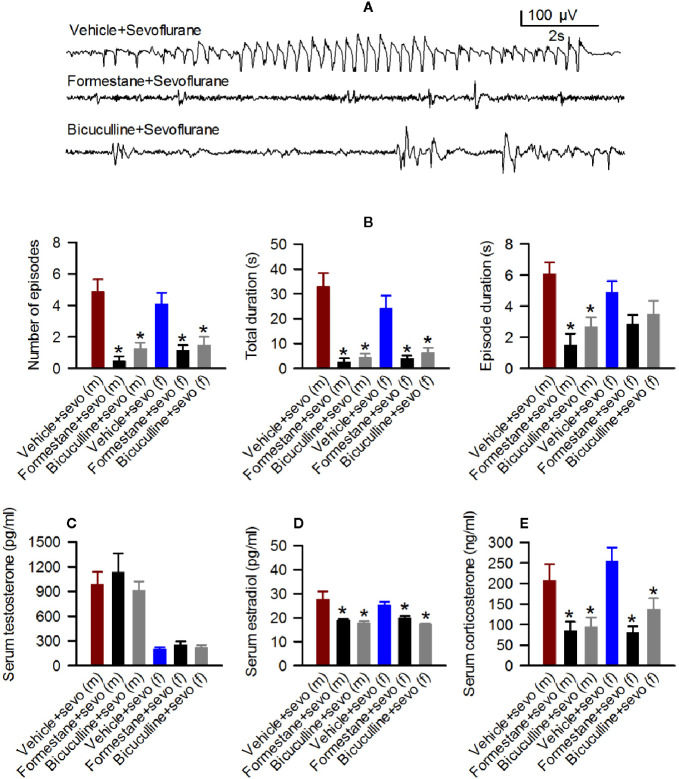
Effects of bicuculline and formestane on sevoflurane-caused electroencephalogram (EEG)-detectable seizures and increases in serum levels of testosterone, estradiol and corticosterone in postnatal day (P) 5 rats. **(A, B)** Effect of bicuculline and formestane on sevoflurane-caused seizures in male and female rats. **(A)** examples of EEGs in sevoflurane-anesthetized P5 male rats that received vehicle, formestane, or bicuculline 30 min prior to initiation of sevoflurane anesthesia. **(B)** Histograms showing parameters of EEG-detectable seizures in male and female rats exposed to sevoflurane that received as pretreatment vehicle, bicuculline, or formestane. Data are means ± SEM from 10 males in the Vehicle + Sevo group, 8 males in the Bicuculline + Sevo group and in the Formestane + Sevo group, 9 females in the Vehicle + Sevo o group, 8 females in the Bicuculline + Sevo group, and 6 females in the Formestane + Sevo group. Animals in the Vehicle + Sevo groups are the same as in **Figure 2**. *P < 0.05 vs. the Vehicle + Sevo group in the respective sex. **(C–E)** Plots showing serum levels of testosterone, estradiol, and corticosterone in blood samples collected after completion of EEG recordings as in **(B)** Data are means ± SEM from six rats/group, five rats/group for estradiol measurements, except six males group in the Vehicle + Sevo group. *P < 0.05 vs. the Vehicle + Sevo group in the respective sex. The tissue samples in the Vehicle + Sevo groups are from the same animals as in **Figures 2** and **3**.

Next, we studied sevoflurane-induced changes in EEG-detectable seizures, serum levels of T, E2 and corticosterone and hypothalamic expressions of *aromatase*, *Er*α, *Er*β, *Nkcc1*, and *Kcc2* in rats that were pretreated with the GABA_A_R antagonist bicuculline or the inhibitor of E2 synthesis formestane prior to the initiation of sevoflurane anesthesia. The number of episodes (F_(2,23)_ = 18.788, *P* < 0.001), the total duration (F_(2,23)_ = 22.505, *P* < 0.001), and the duration of a single episode (F_(2,23)_ = 12.103, *P* < 0.001) of EEG-detectable seizures caused by sevoflurane were significantly diminished in the pretreated male rats (**Figures 4A, B**). Specifically, compared with male rats pretreated with the vehicle (the Vehicle + Sevo group), the total duration (*P* < 0.001, both the Bicuculline + Sevo and Formestane + Sevo groups), the number of episodes (*P* < 0.001, both the Bicuculline + Sevo and Formestane + Sevo groups), and the duration of a single episode (*P* < 0.001, the Formestane + Sevo group; *P* = 0.004, the Bicuculline + Sevo group) were reduced in rats pretreated with either agent. Also, there were between-subjects effects of pretreatments prior to exposure to sevoflurane on the total duration (F_(2,20)_ = 9.496, *P* = 0.001) and the number of episodes (F_(2,20)_ = 8.107, *P* = 0.003), but not on the duration of a single episode (F_(2,20)_ = 1.930, *P* = 0.171) of EEG-detectable seizures caused by sevoflurane in female rat pups (**Figure 4B**). Again, significant reductions of the total duration (*P* = 0.004, both the Bicuculline + Sevo and Formestane + Sevo groups) and the number of episodes (*P* = 0.007, the Formestane + Sevo group; *P* = 0.006, the Bicuculline + Sevo group) were observed in female rats pretreated with bicuculline or formestane.

There were no between-subjects effects of bicuculline or formestane on the serum levels of T in males (F_(2,15)_ = 0.459, *P* = 0.641; **Figure 4C**) and females (F_(2,15)_ = 0.706, *P* = 0.509; **Figure 4C**). In contrast to serum levels of T, there were between-subjects effects of the pretreatments on serum levels of E2 in males (F_(2,13)_ = 6.349, *P* = 0.012; **Figure 4D**). Pairwise multiple comparison analysis showed that bicuculline (*P* = 0.020 vs. the Vehicle + Sevo group) and formestane (*P* = 0.029 vs. the Vehicle + Sevo group) reduced serum levels of E2 in male rat pups. Similarly, effects of bicuculline and formestane on serum levels of E2 were found in females (F_(2,12)_ = 17.842, *P* < 0.001; **Figure 4D**). Both bicuculline (*P* < 0.001 vs. the Vehicle + Sevo group) and formestane (*P* = 0.005 vs. the Vehicle + Sevo) reduced serum levels of E2 in female rat pups. Pretreatments with bicuculline or formestane had significant effects on serum levels of corticosterone increased by sevoflurane anesthesia in males (F_(2,15)_ = 5.543, *P* = 0.016) and in females (F_(2,15)_ = 11.780, *P* < 0.001; **Figure 4E**). Compared to the Vehicle + Sevo group, both bicuculline (*P* = 0.029, males; *P* = 0.012, females) and formestane (*P* = 0.027, males; *P* < 0.001, females) reduced serum levels of corticosterone (**Figure 4E**).

**Figure 5:**
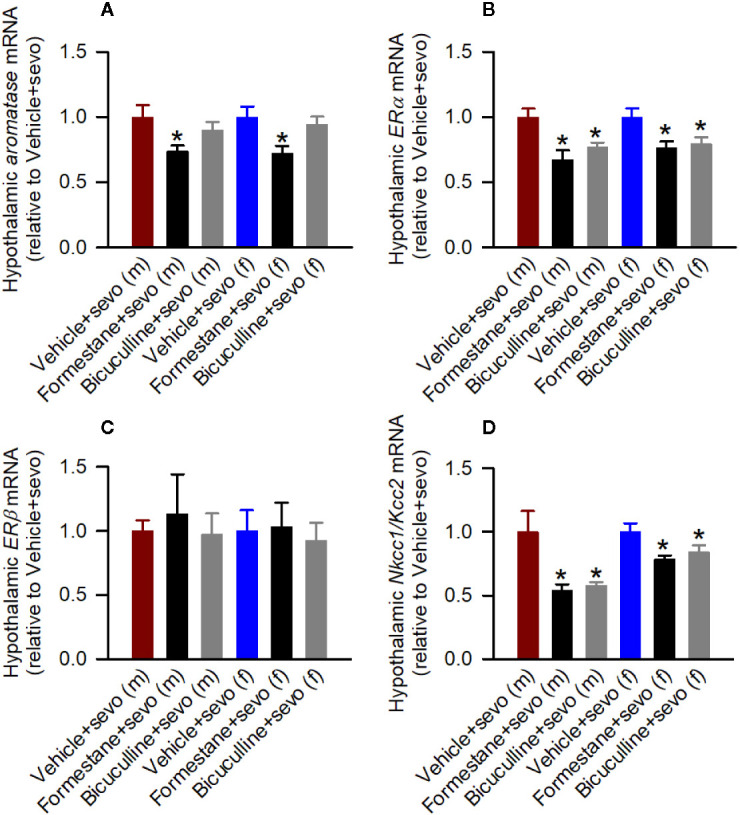
Effects of bicuculline and formestane on sevoflurane-induced changes in gene expressions in the hypothalamus of postnatal day (P) 5 rats. Brain tissue samples were collected from male and female rats immediately after completion of electroencephalography recordings in sevoflurane-anesthetized P5 rats that received vehicle (the Vehicle + Sevo group), formestane (the Formestane + Sevo group), or bicuculline (the Bicuculline + Sevo group) 30 min prior to initiation of sevoflurane anesthesia. **(A–D)** The respective levels of *aromatase* mRNA, estrogen receptor α (*Er*α) mRNA, estrogen receptor β (*Er*β) mRNA, Na^+^-K^+^-Cl^−^ cotransporter *(Nkcc1)/*K^+^-Cl^−^ cotransporter (*Kcc2*) mRNA ratios in the hypothalamus of male and female rats. Data are means ± SEM from six rats/group. *P < 0.05 vs. the sevoflurane group in the respective sex. The tissue samples in the Vehicle + Sevo groups are from the same animals as in **Figure 3**.

In the hypothalamic gene expression measurements, there was a statistically significant between-subjects effect of pretreatments on the hypothalamic levels of *aromatase* mRNA in males (F_(2,15)_ = 3.893, *P* = 0.043; **Figure 5A**) and females (F_(2,15)_ = 4.949, *P* = 0.022; **Figure 5A**). Pairwise multiple comparison analysis showed that compared to the Vehicle + Sevo group, formestane (*P* = 0.015, males; *P* = 0.009, females) but not bicuculline (*P* = 0.317, males, *P* = 0.557, females) ameliorated sevoflurane-induced increase in *aromatase* expression. Similarly, there was a statistically significant between-subjects effect of pretreatments on the hypothalamic levels of *Erα* mRNA in males (F_(2,15)_ = 8.176, *P* = 0.004; **Figure 5B**), and females (F_(2,15)_ = 5.208, *P* = 0.019; **Figure 5B**), but not on the hypothalamic levels of *Erβ* mRNA in males (F_(2,15)_ = 0.168, *P* = 0.847; **Figure 5C**) and females (F_(2,15)_ = 0.107, *P* = 0.899; **Figure 5C**). Pairwise multiple comparison analysis showed that compared to the Vehicle + Sevo group, formestane (*P* = 0.004, males; *P* = 0.030, females) and bicuculline (*P* = 0.028, males, *P* = 0.039, females) ameliorated the sevoflurane-induced increase in *Erα* expression.

There was a statistically significant between-subjects effect of pretreatments with formestane and bicuculline on the hypothalamic *Nkcc1*/*Kcc2* mRNA ratios in males (F_(2,15)_ = 6.487, *P* = 0.009; **Figure 5D**) and in females (F_(2,15)_ = 4.578, *P* = 0.028; **Figure 5D**). The *Nkcc1*/*Kcc2* mRNA ratios compared with the Vehicle + Sevo group were decreased by pretreatments with bicuculline or formestane in males (*P* = 0.019, the Bicuculline + Sevo group; *P* = 0.016, the Formestane + Sevo group) and in females (*P* = 0.049, the Bicuculline + Sevo group; and *P* = 0.001, the Formestane + Sevo group).

### Effects of Pretreatments With Estrogen Receptor Antagonists

**Figure 6:**
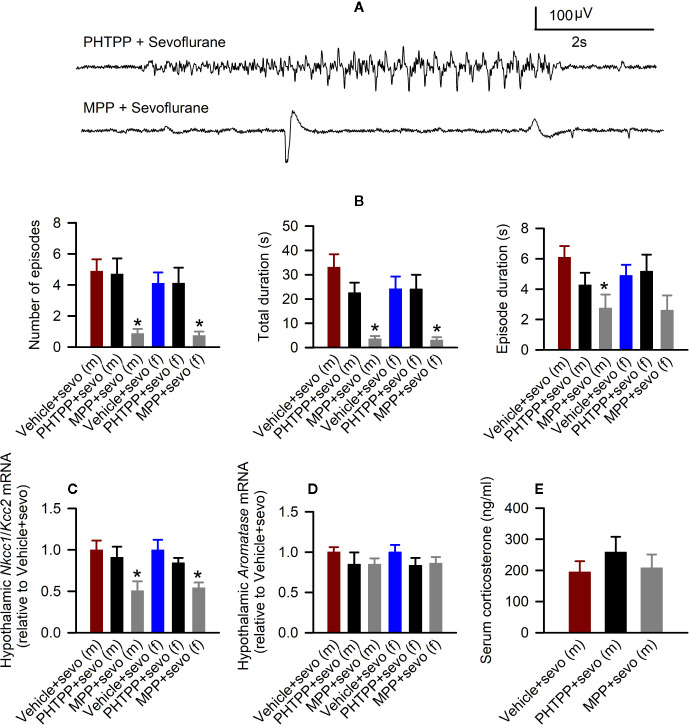
Effects of the estrogen receptor α (ERα) and β (ERβ) antagonists on sevoflurane-induced abnormalities in postnatal day (P) 5 rats. **(A, B)** Effects of the ERα antagonist MPP and ERβ antagonist PHTPP on sevoflurane-caused electroencephalogram (EEG)-detectable seizures in male and female rats. **(A)** examples of EEGs in sevoflurane-anesthetized P5 male rats that received MPP (the MPP + Sevo group), or PHTPP (the PHTPP + Sevo group) 30 min prior to the initiation of sevoflurane anesthesia. **(B)** Histograms showing parameters of EEG-detectable seizures in male and female rats exposed to sevoflurane that received as pretreatment vehicle (the Vehicle + Sevo group), MPP (the MPP + Sevo group), or PHTPP (the PHTPP + Sevo group). Data are means ± SEM from 10 males in the Vehicle + Sevo group, 8 males in the MPP + Sevo group, 7 males in the PHTPP + Sevo group, 9 females in the Vehicle + Sevo group, 8 females in the MPP + Sevo group, and 8 females in the PHTPP + Sevo group. Animals in the Vehicle + Sevo groups are the same as in **Figure 2**. *P < 0.05 vs. the Vehicle + Sevo group in the respective sex. **(C)** The Na^+^-K^+^-Cl^−^ cotransporter *(Nkcc1)/*K^+^-Cl^−^ cotransporter (*Kcc2*) mRNA ratios in the hypothalamus of male and female rats. Data are means ± SEM from 6 rats/group. *P < 0.05 vs. the Vehicle + Sevo group in the respective sex. The mRNA levels of aromatase in the male and female hypothalamus **(D)** and serum levels of corticosterone in males **(E)**. Data are means ± SEM from six rats/group. *P < 0.05 vs. the Vehicle + Sevo group in the respective sex. The tissue samples in the Vehicle + Sevo groups are from the same animals as in **Figures 2** and **3** .

In males, there were statistically significant effects of pretreatments with estrogen receptor antagonists on the total durations of sevoflurane-induced seizures (F_(3,22)_ = 12.824, *P* < 0.001; **Figures 6A, B**), the number of seizure episodes (F_(3,22)_ = 9.614, *P <*0.001; **Figure 6B**), and the episode’s duration (F_(3,22)_ = 4.577, *P* = 0.022; **Figure 6B**). Pairwise multiple comparison analysis showed that compared with the Vehicle + Sevo group, only the ERα antagonist MPP reduced the total durations of seizures (*P* < 0.001, the MPP + Sevo group; and *P* = 0.097, the PHTPP + Sevo group), the number of seizure episodes (*P* = 0.002, the MPP + Sevo group; *P* = 0.859, the PHTPP + Sevo group) and the episode’s duration (*P* = 0.019, the MPP + Sevo group; P = 0.244, the PHTPP + Sevo group). Similarly, in females there were statistically significant effects of pretreatments with estrogen receptor antagonists on the total durations of sevoflurane-induced seizures (F_(3,22)_ = 6.918, *P* = 0.005; **Figure 6B**) and on the number of seizure episodes (F_(3,22)_ = 47.277, *P* = 0.004; **Figure 6B**), but not on the episode’s duration (F_(3,22)_ = 2.291, *P* = 0.125; **Figure 6B**). Again, pairwise multiple comparison analysis showed that compared with the Vehicle + Sevo group only the ERα antagonist MPP reduced the total durations of seizures (*P* = 0.010, the MPP + Sevo group; and *P* = 0.988, the PHTPP + Sevo group) and the number of episodes (*P* = 0.008, the MPP + Sevo group; and *P* = 0.989, the PHTPP + Sevo group).

Also, there was a statistically significant between-subjects effect of pretreatments with estrogen receptor antagonists on the hypothalamic *Nkcc1*/*Kcc2* mRNA ratios in males (F_(2,15)_ = 4.909, *P* = 0.023; **Figure 6C**) or in females (F_(2,15)_ = 7.188, *P* = 0.006; **Figure 6C**). Pairwise multiple comparison analysis showed that compared with the sevoflurane group, only the ERα antagonist MPP ameliorated the effects of sevoflurane on the hypothalamic *Nkcc1*/*Kcc2* mRNA ratios in males (*P* = 0.030, the MPP + Sevo group; and *P* = 0.600, the PHTPP + Sevo group, **Figure 6C**) and females (*P* = 0.006, the MPP + Sevo group; and *P* = 0.220, the PHTPP + Sevo group; **Figure 6C**).

Despite the ameliorating effects of pretreatments with formestane on sevoflurane-induced increases in the serum levels of corticosterone (**Figure 4E**) and hypothalamic expression of aromatase (**Figure 5A**), pretreatments with either MPP or PHTP did not alter these effects of sevoflurane (**Figures 6D, E**). To test whether the GPER, which is expressed in the hypothalamus, hippocampus, and cortex (25) can be involved in the mediation of these effects of sevoflurane, we have studied the effects of pretreatments with the GPER antagonist G-15 on sevoflurane-caused seizures and increases in serum levels of corticosterone in male rats. Compared with the Vehicle + Sevo group, pretreatments with G-15 had no effect on the total durations of sevoflurane-induced EEG-detectable seizures (33.1 ± 5.3 s, the Vehicle + Sevo group (n = 10), and 31.0 ± 10.1 s (n = 7), the G15 + Sevo group, t_(15)_ = 0.200; *P* = 0.844), as well as on the sevoflurane-caused increase in the serum levels of corticosterone (192.232 ± 24.225 ng/ml, the Vehicle + Sevo group, and 192.205 ± 27.253 ng/ml, the G15 + Sevo group, n = 6/group, t_(10)_ = 0.001; *P* = 0.999). All experimental findings are summarized in **Table 2**.

**Table 2:** Summary of the findings of the effects of sevoflurane and the studied agents (pretreatments) on the effects of sevoflurane in postnatal day 5 male and female rats.

Target	Effects of Sevoflurane on targets shown in the left column									
	Males					Females				
Testosterone	Increased (↑)					No effect (0)				
Estradiol	Increased (↑)					Increased (↑)				
Corticosterone	Increased (↑)					Increased (↑)				
Seizures	Increased (↑)					Increased (↑)				
Nkcc1 mRNA	Increased (↑)					Increased (↑)				
Kcc2 mRNA	Decreased (↓)					Decreased (↓)				
Nkcc1/Kcc2 mRNA	Increased (↑)					Increased (↑)				
Aromatase mRNA	Increased (↑)					Increased (↑)				
Erα mRNA	Increased (↑)					Increased (↑)				
Erβ mRNA	No effect (0)					No effect (0)				
Target	**Effects of pretreatments on the effects of Sevoflurane shown above**									
	Pretreatments in males					Pretreatments in females				
	Bicuculline	Formestane	MPP	PHTPP	G-15	Bicuculline	Formestane	MPP	PHTPP	G-15
Testosterone	0	0				0	0			
Estradiol	↓	↓				↓	↓			
Corticosterone	↓	↓	0	0	0	↓	↓			
Seizures	↓	↓	↓	0	0	↓	↓	↓	0	0
Nkcc1/Kcc2 mRNA	↓	↓	↓	0	0	↓	↓	↓	0	0
Aromatase mRNA	0	↓	0	0	0	0	↓	0	0	0
Erα mRNA	↓	↓				↓	↓			
Erβ mRNA	0	0				0	0			

↑ and ↓ show a significant increase and decrease, respectively, caused by sevoflurane when compared to the Control group and by a pretreatment + sevoflurane compared with the Vehicle + Sevoflurane group. 0 means no significant effect and empty cells mean no measure was conducted. Testosterone, estradiol, and corticosterone were measured in serum. The mRNA levels of aromatase, aromatase gene; Erα, estrogen receptor α gene; Erβ, estrogen receptor β gene; Nkcc1, Na^+^-K^+^-Cl^−^ cotransporter gene; Kcc2, K^+^-Cl^−^ cotransporter gene, were measured in the hypothalamus. See text for details.

## Discussion

The novel findings of this study are that during the sensitive period, when T/E2 are known to program permanent changes in the rodent brain (organizational effects), sevoflurane administered to neonatal rats increases the systemic levels of T in males, while it elevates the levels of E2 and upregulates crucial components of the E2 and GABA_A_R signaling pathways in both sexes. The findings of this study indicate that the sevoflurane-increased E2 levels, which are independent of the sevoflurane-increased systemic levels of T, are essential to mediate sevoflurane’s acute adverse effects, at least as they relate to measured gene expressions, EEG-detectable seizures, and stress-like corticosterone responses. Thus, despite the sevoflurane-caused increase in serum T levels in males, all other acute adverse effects of sevoflurane were similar in males and females. All of these acute adverse effects except the sevoflurane-caused increase in the systemic levels of T in males were deterred by pretreatments with the E2 synthesis inhibitor formestane.

Our findings, along with data in the literature, suggest that the brain is the source of heightened systemic levels of E2 in male and female pups anesthetized with sevoflurane. This possibility is consistent with the notion that the ovary, the main source of systemic E2 in females, is quiescent at this age (25, 26) and that both male and female rat pups have similar serum levels of E2 during this age period (38). Also, data in the literature show that removal at birth of the peripheral steroidogenic organs does not reduce the systemic levels of E2 and does not change the brain levels of E2 in rat pups (38). Our finding that the sevoflurane-induced increases in serum levels of E2 in both sexes, but not the sevoflurane-induced increases in serum levels of T in males, were reduced by pretreatments with bicuculline further supports the idea that there are different sources of the sevoflurane-induced increases in serum levels of E2 and T. Although bicuculline is considered poorly permeable through the blood-brain barrier, data in the literature (36) and our findings of the alleviating effects of bicuculline on sevoflurane-caused EEG-detectable seizures and changes in the hypothalamic expressions of estrogen receptors and Cl^−^ co-transporters suggest that bicuculline methiodide induces central effects in neonatal rats. The latter may include the bicuculline methiodide-caused reduction in the systemic levels of E2 in sevoflurane-exposed rat pups. Finally, the sevoflurane-induced saturating levels of E2 synthesized *de novo* in the brain may be a reason for the lack of additive effects of E2 that can be synthesized through the aromatization of systemic T in the brain in male pups. All of these findings suggest that sevoflurane-increased levels of E2, which can be detected as elevated levels in the serum, are formed by E2 that is synthesized in the brain *de novo*. In future studies, it will be important to confirm or refute this possibility by measuring E2 and T levels in different regions of the brain in male and female pups anesthetized with sevoflurane.

If it is further confirmed that E2 synthesized in the brain *de novo*, but not E2 synthesized from systemic T, is involved in the mediation of the acute adverse effects of sevoflurane, this finding may be important for understanding the mechanisms of the developmental effects of sevoflurane. Moreover, it may be important for investigating the fundamental mechanisms of brain development and brain sexual differentiation in particular. It is known that T exerts lasting organizational effects in developing rodent brains (brain masculinization) primarily through its derivative E2 (23, 26). However, the relative roles of E2 synthesized from testis-derived T and E2 synthesized from T in the brain *de novo* are not fully understood (26, 38). Our findings suggest that at least in sevoflurane-anesthetized neonatal rats, the levels of E2 synthesized in the brain *de novo* may be high enough that the brain’s E2 synthesized from testis-derived T does not induce additional acute effects.

The sex hormone-regulated perinatally organized neurocircuitry is activated by the adult steroid hormone environment to express sex-appropriate behavior and physiology, including stress responsivity (39, 40). We have previously demonstrated that adult rats neonatally exposed to sevoflurane or other anesthetics that act through GABA_A_Rs not only have an elevated *Nkcc1*/*Kcc2* mRNA ratio in the hypothalamus and hippocampus, but also exhibit behavioral deficiencies and exacerbated HPA axis responses to stress (8–12). These effects are more pronounced in males. The sevoflurane-induced changes in the systemic levels of T during the early postnatal period may contribute to the anesthetic-induced long-term sex-dependent abnormalities. Notably, although testis-produced T initiates organizational effects in male brain primarily through its aromatized metabolite E2 and subsequent activation of ERα, the androgen receptor-mediated effects of T are also required for T’s organizational effects (41, 42). The organizational T effects in perinatal brain emerge later. The relatively modest short-term effects of T in perinatal brain are followed by dramatic changes in gene expression and behavior in adulthood (43, 44). Of potential relevance, adult male rats, neonatally treated with exogenous T, had significantly decreased circulating T levels and increased aromatase and E2 levels (45). These levels are typically associated with exacerbated HPA axis responses to stress. In future studies, it will be important to investigate in detail the long-term effects of alterations in levels of T and E2 induced by neonatal anesthesia with sevoflurane and other GABAergic anesthetics.

The sevoflurane-induced increases in E2 levels may contribute to acute functional abnormalities induced by sevoflurane in neonatal rats (e.g., EEG-detectable seizures and heightened corticosterone levels) by enhancing excitatory GABA_A_R signaling in the cortex and in the HPA axis, respectively. The GABA_A_R-mediated acute effects of E2 are supported by our previously published findings that exogenous E2 potentiated sevoflurane-caused EEG-detectable seizures in rat pups and increased GABA_A_R-mediated currents in hippocampal slices from these animals (10). The alleviating effects of the pretreatments with bicuculline and formestane on sevoflurane-caused, EEG-detectable seizures and heightened serum levels of corticosterone found in this study further support the GABA_A_R-mediated effects of sevoflurane/E2. The findings in this study, however, suggest a much more complex involvement of E2 in the mediation of sevoflurane-induced adverse effects than just positive modulation of the GABA_A_R-based neurotransmission machinery. Sevoflurane increased not only the levels of E2, but also the mRNA levels of hypothalamic *Er*α and the *Nkcc1*/*Kcc2* ratio. These effects were ameliorated by pretreatments with the inhibitors of GABA_A_R and E2 synthesis. The latter suggests a mediating role of GABA_A_R-dependent E2 synthesis in the transcriptional effects of sevoflurane. Interestingly, the mechanisms mediating sevoflurane-induced changes in gene expression of different components of the E2 pathway may be different. Thus, formestane, but not bicuculline, alleviated the sevoflurane-caused increase in the hypothalamic levels of aromatase. Future studies will be needed to uncover the mediating mechanisms of these effects. This study was not designed to elucidate how changes in gene expressions in each specific experimental group translate to changes in respective protein levels. For that reason, future studies will be needed to determine the relative role of sevoflurane-induced changes in the E2 pathway gene transcriptions in the acute and long-term developmental effects of the anesthetic.

Notably, ERα predominantly, but not exclusively, localizes to the brain regions involved in regulating sexual behavior such as the hypothalamus, whereas ERβ has a broader distribution in the neurons of the hippocampus, cerebral cortex, and amygdala, as well as in microglia and oligodendrocytes (26–29). Nevertheless, the antagonist of ERα, but not ERβ, not only reduced the sevoflurane-increased hypothalamic *Nkcc1*/*Kcc2* mRNA ratio, but also depressed the sevoflurane-induced EEG-detectable seizures, which should predominantly reflect the brain’s cortical activity. In addition, sevoflurane induced an increase in the expression of ERα but not ERβ in the hypothalamus of male and female rat pups, pointing to ERα-specific adverse effects of neonatal sevoflurane. An unexpected and unexplained finding was that the antagonist of ERα, MPP, despite having alleviating effects on sevoflurane-induced EEG-detectable seizures, and the increase in hypothalamic *Nkcc1*/*Kcc2* mRNA ratios did not affect sevoflurane-induced changes in serum levels of corticosterone and hypothalamic expression of *aromatase*. Although the aromatase inhibitor formestane alleviated these effects of sevoflurane, they were not sensitive to pretreatments with the antagonists of ERα, ERβ, and GPER. The latter is consistent with our previously reported observation that the nonselective estrogen receptor antagonist ICI182780 was also ineffective in preventing sevoflurane-caused increases in serum levels of corticosterone in neonatal rats (10). We studied the effects of the ERα, ERβ, or GPER selective antagonists using a single dose for each compound. Therefore, the possibility that treatment with higher doses of the ERα selective antagonist may still be effective against sevoflurane-induced changes in serum corticosterone levels and *aromatase* expression cannot be ruled out. However, the alleviating effects of MPP at this dose on sevoflurane-caused seizures and increases in the *Nkcc1*/*Kcc2* mRNA ratio reduces the probability that higher doses of MPP will be effective against sevoflurane-caused increases in serum corticosterone levels and hypothalamic expression of aromatase.

In conclusion, this study provides evidence that sevoflurane increases systemic levels of T in male rat pups and E2 levels in males and females during the sensitive period of the organizational effects of T and E2. Our findings demonstrate that sevoflurane-increased levels of E2, which are independent of sevoflurane-increased systemic levels of T, are sufficient to mediate sevoflurane-induced corticosterone secretion, EEG-detectable seizures, and complex alterations in expressions of genes that are crucial for the E2 and GABA_A_R signaling pathways. In this study, we provided evidence that a specific estrogen receptor, ERα, is involved in the mediation of E2-dependent acute adverse effects of sevoflurane in neonatal rats.

## Data Availability Statement

All relevant data is contained within the article: The original contributions presented in the study are included in the article; further inquiries can be directed to the corresponding authors.

## Ethics Statement

The animal study was reviewed and approved by: All experimental procedures were approved by the University of Florida Institutional Animal Care and Use Committee.

## Author Contributions

AM and JZ conceptualized and designed the study. NL, YL, NX, and LL acquired data and performed data analysis. Analyses and interpretation of the data and writing of the article were performed by NL, AM, JZ, LJ, TM, and NG. All authors contributed to the article and approved the submitted version.

## Funding

Supported by the Natural Science Foundation of China (Nos. 81771149 and U1704165 to JZ), the National Institutes of Health, Bethesda, MD, USA (Nos. R01NS091542, R01NS091542-S and R56HD102898 to AM), the I. Heermann Anesthesia Foundation (LJ), and the Jerome H. Modell, M.D., F.A.H.A. Endowed Professorship (NG).

## Conflict of Interest

The authors declare that the research was conducted in the absence of any commercial or financial relationships that could be construed as a potential conflict of interest.
